# La bride congénitale: une cause rare d'occlusion néo-natale

**Published:** 2012-12-03

**Authors:** Mohamed Rami, Youssef Bouabdallah

**Affiliations:** 1 Service de chirurgie pédiatrique, CHU Hassan II, Fès, Maroc

**Keywords:** Occlusion intestinale, bride congénitale, nouveau né, syndrôme occlusif, neonatal occlusion, congenital band, newborn, occlusive syndrome

## Images in Medicine

L'occlusion de l'intestin grêle sur bride congénitale (OGBC) est une pathologie rare qui résulte de l'accolement anormal des feuillets péritonéaux donnant naissance à une bride congénitale. Elle pose un réel problème diagnostique, vu qu'elle survient en général chez un patient jamais opéré auparavant, pouvant aboutir à une nécrose intestinale si elle est opérée tardivement. Le diagnostic se fait alors en per opératoire. Nous rapportons le cas d'un nouveau né de 13 jours, admis pour un syndrôme occlusif depuis moins de 48 h, alors qu'il était normal jusque là. Avec à l'examen abdominal une distension abdominale, avec une épreuve à la sonde négative. La radiographie thoraco-abdominale a montré une distension digestive importante sans niveau hydro-aérique. Un lavement à la gastrographine n'a pas montré de disparité de calibre, et l’échographie abdominale était normale. Après une bonne réanimation hydroélectrolytique, le patient a été opéré en urgence, l'exploration chirurgicale a trouvé une distension grêlique en amont d'une zone inflammée située à 5 anses de la valvule iléo-caecale, avec des brides grêlo-grêliques à ce niveau. Le geste a consisté en une libération des brides avec résection puis anastomose de la zone inflammée qui était sténosée. L'alimentation a été reprise après 3 jours, les suites étaient simples. Le recul est de 18 mois.

**Figure 1:**
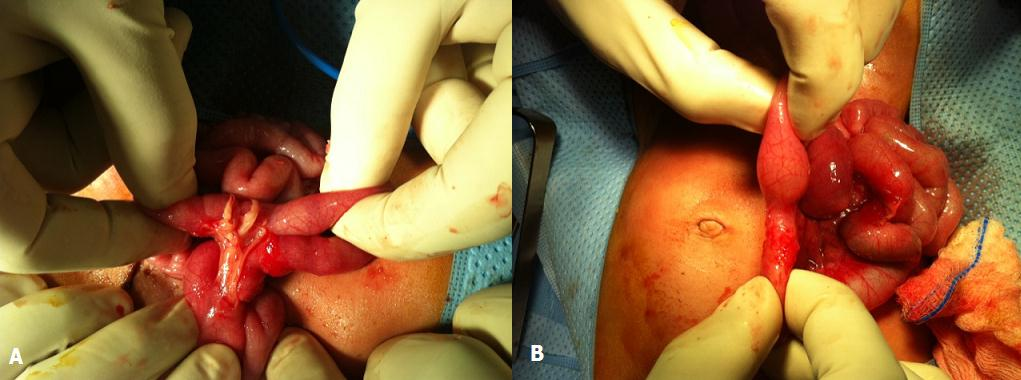
A: bride congénitale grêlo-grêlique avec inflammation locale; B: aspect sténosé après libération de la bride

